# Evaluation of the correlation between fetuin-B levels and essential hypertension: a cross-sectional study

**DOI:** 10.1530/EC-23-0172

**Published:** 2023-11-08

**Authors:** Tao Gao, Rui Liu, Chunli Li, Xinglin Chu, Qiao Guo, Dazhi Ke

**Affiliations:** 1 Department of General Practice, The Second Affiliated Hospital of Chongqing Medical University, Chongqing Medical University, Chongqing, China; 2 Department of Oncology. The Second Affiliated Hospital of Chongqing Medical University, Chongqing Medical University, Chongqing, China; 3 Institute of Life Sciences, Chongqing Medical University, Chongqing, China

**Keywords:** fetuin-B, cytokines, essential hypertension, lipid metabolism

## Abstract

**Background:**

Fetuin-B, a cytokine that regulates lipid metabolism, has recently been linked to cardiovascular diseases such as coronary heart disease. In this study, we discussed the relationship between fetuin-B and essential hypertension.

**Method:**

A bioinformatics analysis of fetuin-B was performed. A total of 206 with essential hypertension and 180 age- and-sex-matched healthy subjects were enrolled. Plasma fetuin-B, endothelin 1 (ET-1), nitric oxide (NO), and adiponectin (ADI) levels were measured using ELISA kits.

**Results:**

Bioinformatics analysis has revealed that fetuin-B plays an important role in pathways such as lipid metabolism. Compared with healthy subjects, serum fetuin-B levels in patients with essential hypertension were significantly increased. Correlation analysis showed that the serum fetuin-B level was positively correlated with systolic blood pressure (SBP), diastolic blood pressure, body mass index, fat percentage* in vivo*, waist–hip ratio, intima–media thickness, low-density lipoprotein cholesterol (LDL-C), glutamyltranspeptidase, alanine transaminase, albumin, fasting blood glucose (FBG), glycated hemoglobin, and ET-1 in the overall study subjects (all *P* < 0.05) and negatively correlated with HDL-C, ADI, and NO (all *P* < 0.05). Multivariate linear regression analysis showed that SBP, FBG, LDL-C, ADI, and ET-1 were independent factors affecting serum fetuin-B. A binary logistic regression analysis showed that fetuin-B was an independent risk factor for primary hypertension (odds ratio: 1.060, 95% CI: 1.034–1.086, *P* < 0.001). Receiver operating characteristic curve analysis was used to evaluate the predictive value of fetuin-B for primary hypertension, and the optimal cutoff point was 83.14 μg/mL (sensitivity 77.4%, specificity 63.3%) (area under the curve) = 0.7738, 95% CI 0.7276–0.8200, *P* < 0.001).

**Conclusion:**

Elevated fetuin-B levels are associated with an increased risk of essential hypertension.

## Introduction

Fetuin-B is synthesized and secreted by the liver, widely exists in blood circulation and in the kidneys, lungs, and ovaries, and is encoded by FETUB gene (1). This gene is distributed in eight exons of human chromosome 3q27.3 (2), which is consistent with the susceptible sites for metabolic syndrome (MS). It has a wide range of physiological functions such as regulating blood glucose and lipid metabolism and inhibiting tissue calcification (3, 4, 5). Previous studies have shown that fetuin-B is closely related to obesity and metabolic syndrome and can lead to lipid metabolism disorders. In a clinical case–control study, it was found that people with nonalcoholic fatty liver disease (NAFLD) had an increased concentration of serum fetuin-B (6). At present, clinical studies have shown that fetuin-B may increase the risk of cardiovascular events (7). Lipid metabolism disorders and overweight are risk factors of hypertension. The researchers knocked down the FETUB gene of human liver cell lines and used fluorescence quantitative PCR technology to measure the normal levels of gene expression of fatty acid metabolism enzymes. They found that the transcription level of genes encoding fatty acid synthase increased, while the transcription level of genes encoding fatty acid lyase decreased. At the same time, oil red staining and TG content determination techniques were used to further confirmed that the formation of intracellular lipid droplets and the content of triglycerides were significantly increased compared to the control group in knockdown liver cells. In addition, it has been found that the activation of farnesoid X receptor leads to upregulation of gene expression encoding fetuin-B (8). Zhu and other scholars also found that the content of fetuin-B was related to the severity of vascular diseases and speculated that fetuin-B might lead to the occurrence and development of coronary heart disease through inflammation (9). Jung *et al.* found that the expression of fetuin-B in the serum of patients with acute myocardial infarction was increased, and it was also confirmed that fetuin-B is related to the generation of atherosclerotic plaques and promotes the rupture of these plaques (10, 11, 12). Fetuin-B plays an important role in regulating lipid metabolism and can result in lipid abnormalities that increase the risk of hypertension, cardiovascular disease, and endothelial dysfunction. Fetuin-B can cause macrophages to accumulate lipids in a manner that promotes the migration of macrophages and monocytes, thus causing vascular endothelial injury by regulating tumor necrosis factor alpha and interleukin 6 to activate the inflammatory pathway of vascular endothelial cells. Inflammatory cells then enter the blood vessel wall along with LDL molecules because of increased endothelial permeability. LDL is oxidized and taken up by the macrophages, which later become foam cells. This is followed by smooth cell proliferation and neovascularization which ultimately cause the thickening of the blood vessel and plaque formation. Endothelin-1 is an endogenous vasoconstrictor regulator that plays an important role in regulating basal vascular tone and cardiovascular system homeostasis. Nitric oxide (NO) is mainly produced by vascular endothelial cells, which can dilate blood vessels, thereby regulating blood pressure and blood flow distribution; it can also inhibit the expression and release of endothelin 1 (ET-1) to a certain extent. The imbalance between endothelin and NO produced by vascular endothelial cells can cause vasodilation and systolic dysfunction, leading to the occurrence and development of hypertension. However, to date, no study has evaluated whether the function of fetuin-B can induce hypertension, and the correlation between fetuin-B and hypertension remains unclear. Therefore, this study aims to further explore the correlation and mechanism between fetuin-B and hypertension.

## Materials and methods

### Participants and methods

This cross-sectional study enrolled 386 subjects, all of whom were from the Second Affiliated Hospital of Chongqing Medical University. From November 2020 to May 2021, 206 patients with primary hypertension were selected, and 180 healthy adults who had undergone physical examination in the Health Management Center of the Second Affiliated Hospital of Chongqing Medical University were matched with them. The inclusion criteria were patients who met the following diagnostic criteria for essential hypertension: a systolic blood pressure (SBP) ≥140 mmHg and/or diastolic blood pressure (DBP) ≥90 mmHg without the use of antihypertensive drugs when the blood pressure was measured three times on different days. The exclusion criteria were as follows: hypertensive patients currently using antihypertensive drugs and lipid-lowering drugs; those with malignant hypertension or secondary hypertension; those with a fasting blood glucose (FBG) ≥7.0 mmol/L; those with a plasma glucose content ≥11.1 mmol/L 2 h after an oral glucose tolerance test; those with heart diseases; those with chronic or acute infectious diseases; or those with chronic kidney diseases. The study was approved by the Ethics Committee of the Second Affiliated Hospital of Chongqing Medical University, and informed consent was obtained from all research subjects.

### Physical examination and laboratory tests

After an overnight fast for at least 12 h, anthropometric measurements were made, and blood samples were obtained by professionals from all participants. Body measurements (weight, height, waist circumference, hip circumference, blood pressure, and fat percentage *in vivo* (FAT%)) and biochemical indices including FBG, total cholesterol (TC), triglyceride (TG), high-density lipoprotein cholesterol (HDL-C), low-density lipoprotein cholesterol (LDL-C), and glycated hemoglobin (HbA1c) levels were measured by high-performance liquid chromatography. Circulating fetuin-B(JM-1455H1), adiponectin (ADI) and ET-1 concentrations were determined by commercial ELISA kits following the manufacturer’s protocol (Jingmei Engineering, Jiangsu Province, China). For all kits, the interassay and intra-assay coefficients of variation were <12 and < 8%, respectively. The minimum detectable concentration for fetuin-B was 0.1 μg/mL. And the preparation and operation steps of the reagent were performed strictly in accordance with the instructions.

### Bioinformatics analysis

#### Protein–protein interaction network construction

The Search Tool (v11.0) for the Retrieval of Interacting Genes (STRING) database was used to construct the PPI network (13). The species was limited to *Homo sapiens*, the cutoff value of confidence score was set to 0.4, and the interaction of proteins was predicted; thereby, an interaction network diagram of a protein can be constructed.

#### Gene Ontology and Kyoto Encyclopedia of Genes and Genome analysis

We used the clusterProfiler package to perform Gene Ontology (GO) and Kyoto Encyclopedia of Genes and Genome (KEGG) pathway analyses (14, 15, 16). A list of GO and KEGG annotation terms were thus obtained. Furthermore, we categorized all genes to the biological process (BP), cellular component (CC), and molecular function (MF) GO categories. *P* < 0.05 indicated statistical significance in the case of GO and KEGG terms.

### Statistical analysis

All statistical analyses were conducted using the Windows version of SPSS 26.0 (SPSS Inc.) and R software (version 4.1.3; R Foundation for Statistical Computing, Vienna, Austria). The Kolmogorov–Smirnov test was used to determine the normality distribution of continuous variables. The results are expressed as the mean ± s.d. or median (25th–75th percentiles). Categorical variables are expressed as frequencies and percentages. Non-normally distributed data were converted by a logarithmic transformation before analysis. A paired *t*-test or an unpaired *t*-test was used for comparisons among groups. Differences among the groups were tested using analysis of variance (ANOVA). Correlational analyses were performed using Pearson’s or Spearman’s correlation tests. Multiple regression analysis was performed to correct the effects of the covariates and evaluate the independent factors. Logistic regression analysis was performed to determine independent predictors of essential hypertension. Restricted cubic splines were used to examine the correlation between plasma fetuin-B levels and the risk of essential hypertension. For all analyses, *P* < 0.05 was considered statistically significant.

## Results

### Bioinformatics analysis

**Figure 1:**
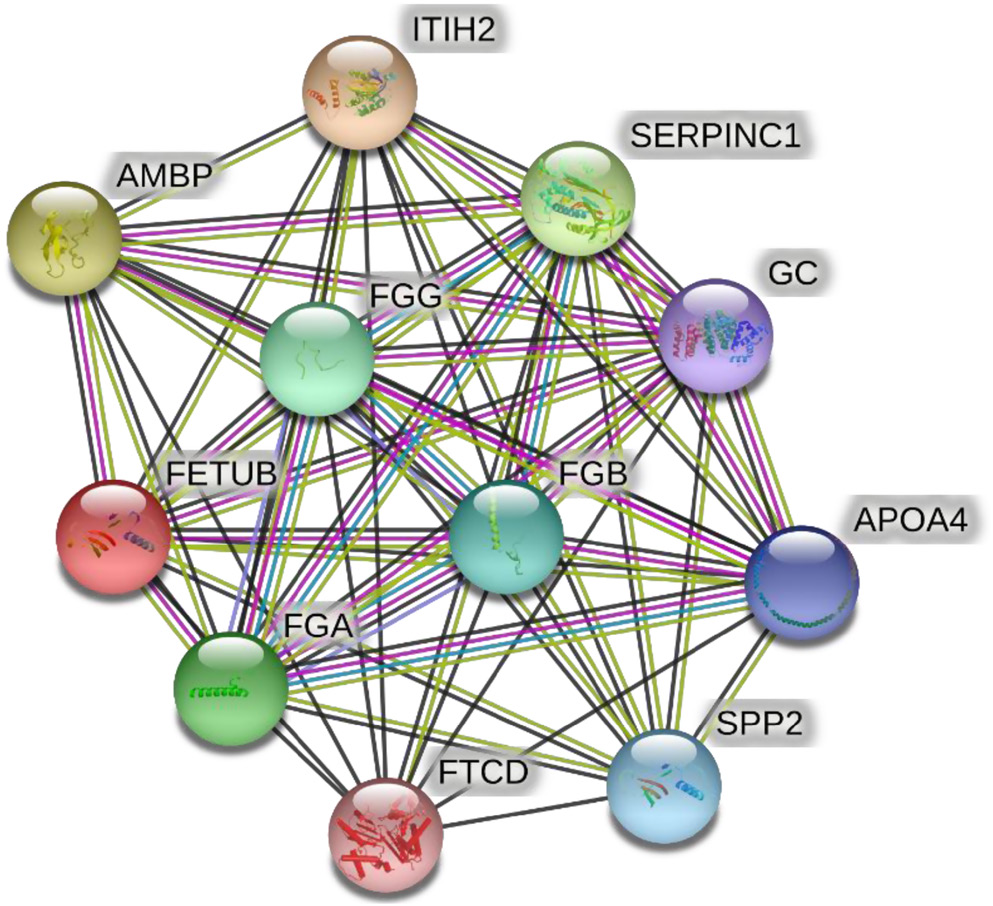
The protein–protein interaction networks of fetuin-B.

**Figure 2:**
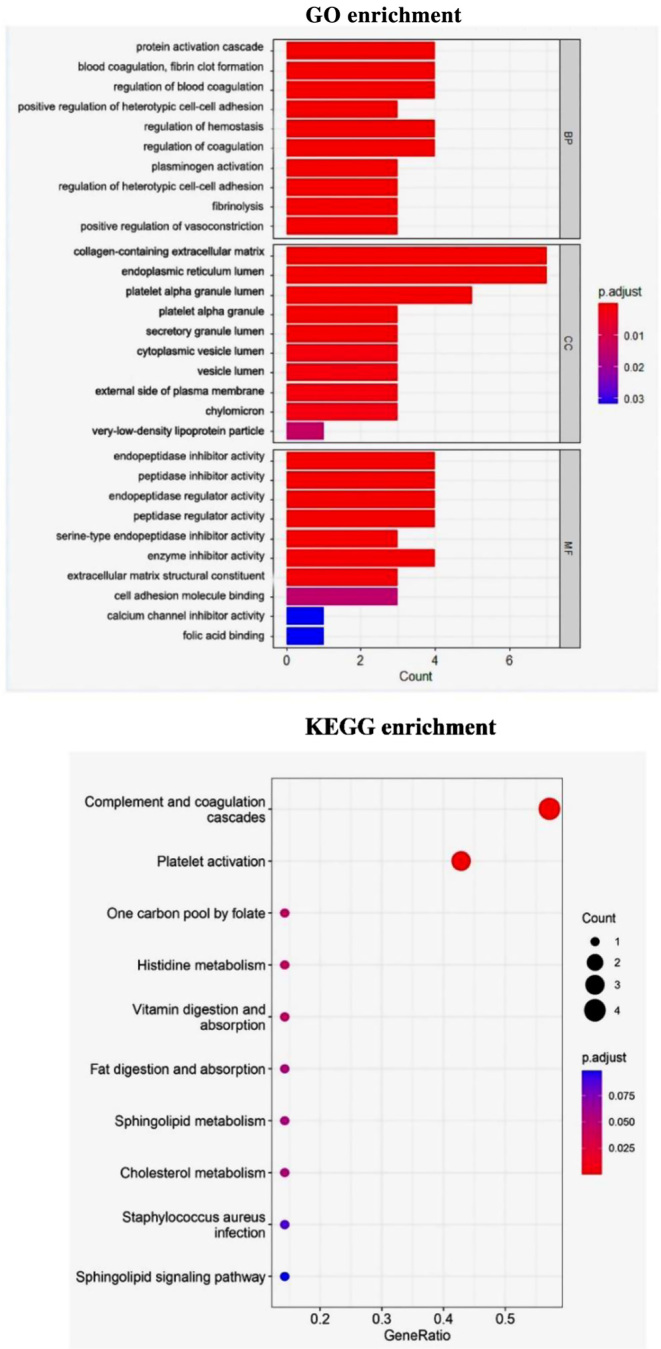
The results of GO and KEGG analysis. Enrichment analysis of fetuin-B interacting proteins in biological process (BP), molecular function (CC), and cellular components (MF); The top 20 significantly enriched pathway terms by clusterProfiler.

According to the confidence score, the top 10 proteins that directly or indirectly interacted with fetuin-B were selected, and the protein obtained was visualized to construct the interaction network diagram of that protein (Fig. 1). We found that the proteins interacting with fetuin-B included polipoprotein A-IV (Apo A-IV), secreted phosphoprotein 24 (SPP24), alpha-trypsin inhibitor heavy chain H2 (ITIH2), alpha-1-microglobulin/bicunin precursor (AMBP), antithrombin-III (SERPINC1), fibrinogen alpha chain (FGA), vitamin D-binding protein (GC), and formyl aminotransferase-cyclodeaminase (FTCD). We obtained unique protein names by downloading the proteins interacting with fetuin-B and removing duplicate proteins. Then, the proteins interacting with fetuin-B were subjected to GO function enrichment analysis from the aspects of BP, CC, and MF aspects in turn. *P* < 0.05 was used as the screening condition, and the one with the highest confidence level (arranged according to the *P*-values) was selected. In terms of BPs, proteins interacting with fetuin-B were mainly involved in protein activation cascade, vasoconstriction, heterotypic cell adhesion, hemorrhage, coagulation, and other processes. In terms of CCs, fetuin-B interacting proteins were mainly enriched in collagen-containing extracellular matrix, endoplasmic reticulum cavity, cytoplasmic vesicle cavity, intracellular protein transport, secretion, and lipid metabolism. In terms of MFs, fetuin-B interacting proteins were mainly involved in regulating endopeptidase inhibition, cell adhesion molecule binding, calcium channel blocking, and other processes (Fig. 2).

In the KEGG pathway enrichment analysis, we used *P* < 0.05 as the screening condition and arranged the *P*-values from large to small. The results showed that the proteins interacting with fetuin-B were mainly involved in complement and coagulation cascade reaction, platelet activation, histidine metabolism, vitamin metabolism and absorption, fat digestion and absorption, sphingolipid metabolism, cholesterol metabolism, and other pathways (Fig. 2).

### Basic laboratory and clinical characteristics

The statistical results are shown in Table 1. A total of 386 subjects were included in this study. There were 206 patients in the essential hypertension group and 180 subjects in the normal control group. There was no significant difference in sex composition, age, and TG, HDL-C, FBG, HbA1c, AST and ALT levels between the two groups. Blood pressure, BMI, carotid intima–media thickness and TC, LDL-C, GGT, ALP, and ET-1 levels were higher in patients with essential hypertension, whereas the ADI and NO levels were lower in healthy controls (all *P <* 0.05).

**Table 1:** Clinical characteristics of study subjects.

Variable		Group		*P*
		NT (*n* = 180)	EH (*n* = 206)	
Sex	M	80 (44.4)	95 (46.1)	0.742
	F	100 (55.6)	111 (53.9)	
SBP (mmHg)		117.34 ± 10.49	171.92 ± 15.69	<0.001
DBP (mmHg)		69.88 ± 7.97	92.68 ± 13.93	<0.001
Age (years)		59.52 ± 6.89	58.70 ± 13.12	NS
BMI (kg/m^2^)		23.05 ± 2.28	24.80 ± 2.38	<0.001
FAT%		28.98 ± 5.32	30.98 ± 5.61	0.002
WHR		0.84 ± 0.08	0.91 ± 0.05	<0.001
IMT		0.63 ± 0.19	0.87 ± 0.21	<0.001
TG (mmol/L)		1.81 ± 0.58	1.86 ± 0.49	NS
TC (mmol/L)		4.75 ± 1.01	5.15 ± 0.92	<0.001
LDL-C (mmol/L)		2.47 ± 0.76	2.77 ± 0.66	<0.001
HDL-C (mmol/L)		1.23 ± 0.30	1.18 ± 0.25	0.074
FBG (mmol/L)		5.68 ± 0.66	5.64 ± 0.49	NS
HbA1c%		5.61 ± 0.46	5.67 ± 0.51	NS
Fetuin-B (μg/mL)		66.46 ± 15.51	76.65 ± 14.11	<0.001
ADI (μg/mL)		26.76 ± 6.17	19.57 ± 3.98	0.009
ET-1 (μg/mL)		97.74 ± 21.95	119.33 ± 22.72	<0.001
NO (μmol/mL)		0.06 ± 0.02	0.04 ± 0.01	<0.001
AST (U/L)		25.54 ± 1.53	25.82 ± 1.60	NS
ALT (U/L)		21.38 ± 0.36	22.21 ± 1.83	NS
ALP (IU/L)		64.13 ± 16.64	70.03 ± 20.86	<0.001
GGT (U/L)		25.90 ± 1.53	26.35 ± 1.75	<0.05
Albumin (g/L)		40.63 ± 0.19	40.87 ± 0.21	<0.001

Data are expressed as the mean with s.d. and median (interquartile). *P*-value for difference between the groups was calculated from Student’s test.

ADI, adiponectin; ALP, alkaline phosphatase; ALT, alanine transaminase; AST, aspartate transaminase; BMI, body mass index; DBP, diastolic blood pressure; ET-1, endothelin 1; FBG, fasting blood glucose; GGT, glutamyltranspeptidase; HbA1c, glycated hemoglobin; HDL-C, high-density lipoprotein cholesterol; IMT, intima–media thickness; LDL-C, low-density lipoprotein cholesterol; NO, nitric oxide; SBP, systolic blood pressure; TC, total cholesterol; TG, triglycerides; WHR, waist–hip ratio.

**Figure 3:**
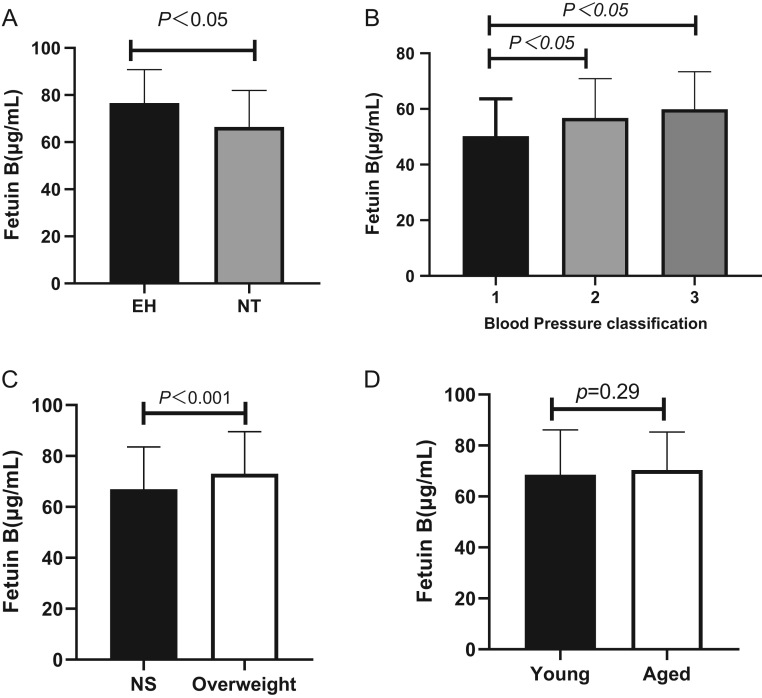
Parameters in hypertensive subjects and healthy controls. (A) The circulating fetuin-B levels in EH and NT group; (B) comparison of the concentration of fetuin-B in different hypertension stage in essential hypertension group; (C) the circulating fetuin-B levels in overweight and NT group; (D) the circulating fetuin-B levels in young and aged groups.

Serum fetuin-B levels in patients with essential hypertension were significantly increased (76.65 ± 14.11 μg/mL vs 66.46 ± 15.51 μg/mL, *P* < 0.001) (Fig. 3A). A subgroup analysis showed that there was no significant difference in the serum fetuin-B level between the elderly group and other age groups (Fig. 3D), while the overweight group had a higher serum fetuin-B concentration than the normal weight group (Fig. 3C). Then, we divided hypertensive patients into three groups according to their blood pressure grades and compared the serum fetuin-B levels between these groups. The results showed that serum fetuin-B levels increased with increases in blood pressure, but there was no significant difference in fetuin-B levels between patients with grade 2 and grade 3 hypertension (Fig. 3B).

**Figure 4:**
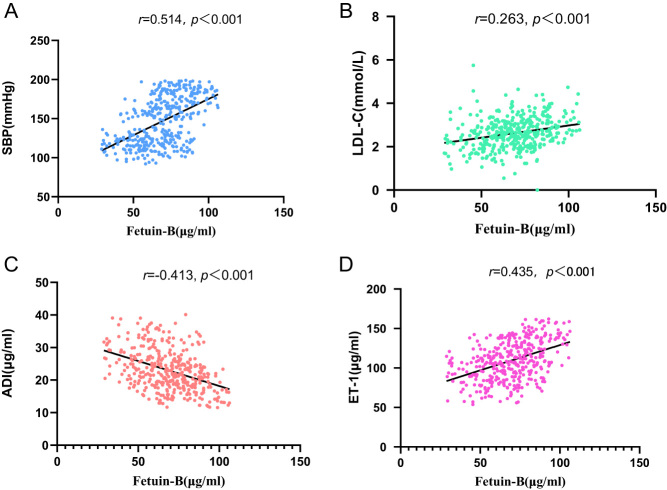
Correlations of plasma fetuin-B levels and SBP (A), LDL-C (B), ADI (C), ET-1 (D) in general population.

As shown in Table 2, in all subjects, serum fetuin-B levels were positively correlated with the levels of SBP (*r* = 0.514, *P* < 0.001) (Fig. 4A), LDL-C (*r* = 0.263,* P* < 0.001) (Fig. 4B), and ET-1 (*r* = 0.435, *P* < 0.01) but negatively correlated with ADI (*r* = −0.413,* P* < 0.001) (Fig. 4C). To further study the relationship between serum fetuin-B and hypertension, we developed a multiple linear regression model. It revealed that TG, LDL-C, ET-1, FBG, and ADI levels were independent variables associated with fetuin-B. The row mean scores test and Cochran–Armitage trend test showed that serum fetuin-B had a significant linear trend with hypertension and was an independent influencing factor of hypertension (Supplementary Table 1, see section on supplementary materials given at the end of this article). A restricted spline curve showed that the incidence of hypertension increased with increases in serum fetuin-B, and serum fetuin-B was a risk factor of hypertension (Supplementary Fig. 1).

**Table 2:** Linear regression analysis of variables associated with circulating fetuin-B levels in study population.

Variable	Simple		Multiple	
	*r*	*P*	*β*	*P*
SBP (mmHg)	0.514	<0.001	0.326	<0.001
DBP (mmHg)	0.370	<0.001		
Age (years)	−0.014	0.781		
BMI (kg/m^2^)	0.265	<0.001		
FAT%	0.138	0.007		
Albumin (g/L)	0.181	<0.001		
GGT (U/L)	0.131	0.01		
ALP (IU/L)	0.047	0.354		
ALT (U/L)	0.161	0.002		
AST (U/L)	0.06	0.237		
WHR	0.232	<0.001		
IMT	0.200	<0.001		
TG (mmol/L)	0.058	0.255		
TC (mmol/L)	0.019	0.704		
LDL-C (mmol/L)	0.263	<0.001	0.118	0.017
HDL-C (mmol/L)	−0.152	0.003		
FBG (mmol/L)	0.279	<0.001	0.170	<0.001
HbA1c%	0.125	0.014		
ADI (μg/mL)	−0.413	<0.001	−0.155	0.002
ET-1 (μg/mL)	0.435	<0.001	0.241	<0.001
NO (μmol/mL)	−0.273	<0.001		

In multiple linear regression analysis, values included for analysis were age, BMI, WHR, FAT%, SBP, FBG, HbA1c, TC, HDL-C, LDL-C, TG, NO, ET-1,AST, ALT, GGT, ALP, and ADI. Spearman’s correlation tests were used in the analysis.

ADI, adiponectin; ALP, alkaline phosphatase; ALT, alanine transaminase; AST, aspartate transaminase; BMI, body mass index; DBP, diastolic blood pressure; ET-1, endothelin 1; FBG, fasting blood glucose; GGT, glutamyltranspeptidase; HbA1c, glycated hemoglobin; HDL-C, high-density lipoprotein cholesterol; IMT, intima–media thickness; LDL-C, low-density lipoprotein cholesterol; NO, nitric oxide; SBP, systolic blood pressure; TC, total cholesterol; TG, triglycerides; WHR, waist–hip ratio.

**Figure 5:**
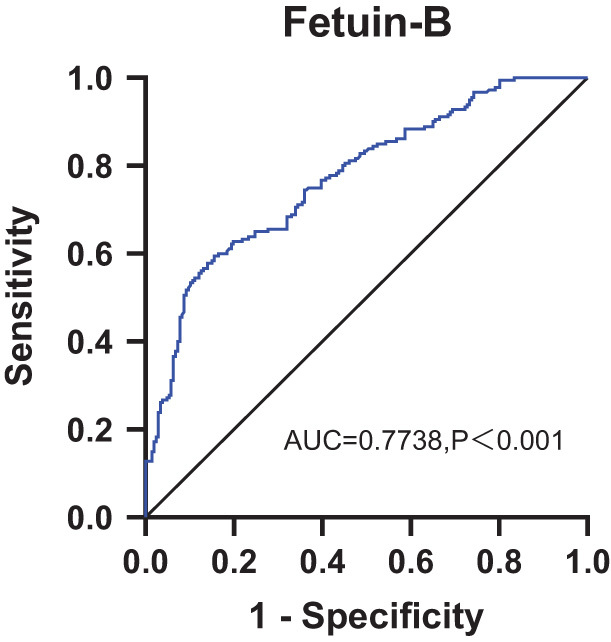
ROC curves analyses were performed for the diagnosis of EH according to the fetuin-B levels.

In the logistic regression analysis, the relationship between the variables and the prevalence of hypertension in 386 subjects was analyzed. Serum fetuin-B was still an independent risk factor for hypertension after adjusting for anthropometric variables including gender, blood lipid levels, FBG, and other factors (Table 3). A receiver operating characteristic (ROC) curve was used to evaluate the value of serum fetuin-B in diagnosing essential hypertension. The results showed that the best cutoff point of fetuin-B in predicting hypertension was 63.14 μg/mL (sensitivity of 77.4%, specificity of 63.3% (area under the curve (AUC) = 0.7738, 95% confidence interval (CI): 0.7276–0.8200, *P* < 0.001) (Fig. 5).

**Table 3:** Odds ratio of circulating feuitn-B level with hypertension by multivariate logistic regression analysis.

Model adjust	EH		
	OR	95% CI	*P*
Model 1	1.075	1.057-1.094	<0.001
Model 2	1.076	1.058-1.094	<0.001
Model 3	1.075	1.051-1.100	<0.001
Model 4	1.060	1.034-1.086	<0.001

Model 1: unadjusted.

Model 2: adjusted for age and gender.

Model 3: adjusted for age, gender, FAT%, WHR, IMT, BMI.

Model 4: age, gender, FAT%, WHR, IMT, BMI, TC, TG, HDL-C, HbA1c%, FBG, LDL-C, AST, ALT, GGT, ALP.

## Discussion

Fetuin-B is a 380-residue glycoprotein and belongs to the cystatin superfamily of cysteine protease inhibitors, which is predominantly synthesized in the liver and secreted into the circulation. Recently, more studies have focused on the relationship between fetuin-B and cardiovascular disease. Serum fetuin-B was elevated in patients with coronary artery disease and carotid artery stenosis, and correlated with the severity of coronary artery disease and carotid plaque burden. However, it is still controversial whether fetuin-B can be used as a biomarker of cardiovascular disease. Furthermore, previous studies did not exclude the effects of comorbidities, liver function, and drugs on the experimental results. Therefore, to avoid these shortcomings, we investigated the association of fetuin-B with hypertension in newly diagnosed hypertensive patients.

After excluding the confounding factors such as liver dysfunction and diabetes mellitus, our study found that the serum fetuin-B levels of patients with essential hypertension were higher than those of healthy controls. In general population, we observed that serum fetuin-B levels were significantly positively correlated with blood pressure and independently correlated with ET-1, an indicator of vascular endothelial dysfunction. Logistic regression analysis suggested that serum fetuin-B was an independent risk factor for essential hypertension. Hypertension is a complex disease with a multifaceted pathogenesis. It is considered to be the result of disturbances in a number of neural, renal, hormonal, and vascular mechanisms, in which impairment of constriction and relaxation of vessels is the main pathophysiologic characteristic. At present, research shows that changes in vascular structure and function are the main factors involved in the pathogenesis of hypertension (17, 18). In our study, we found that the ET-1 level in EH group was significantly higher than the NT group. ET-1 is produced by vascular endothelial cells and has strong vasoconstrictive and angiogenic effects. And increases in serum ET-1 levels can lead to the disruption of the balance of vasoconstriction and vasodilation, which suggests that vascular endothelial function is damaged. Moreover, we observed that there was a significant positive correlation between serum ET-1 and fetuin-B levels. Multiple linear regression analysis suggested that ET-1 was an independent factor correlated with fetuin-B. We calculated the AUC value of ET-1 to be able to compare their discriminatory ability. We found that the values were quite similar between ET-1 and fetuin-B (0.743 vs 0.7738). It seems that there is a possible relationship between these two factors.

In addition, we found that the serum fetuin-B concentration in hypertensive patients was positively correlated with the LDL-C, TG, and TC levels and negatively correlated with the ADI level. Multiple linear regression analysis showed that LDL-C and ADI were independent factors correlated with fetuin-B. This suggests that fetuin-B may be involved in regulating lipid metabolism. We also confirmed this view through bioinformatics analysis. By constructing a PPI network, we identified 10 interacting proteins of fetuin-B. They were enriched in pathways related to glucose and lipid metabolism. Among the identified proteins, AMBP has the functions of immunomodulation, inhibition of inflammatory mediators, antioxidation, and inhibition of smooth muscle contraction (19, 20). SPP2 has the effects of regulation of vascular calcification and anti-inflammation (21, 22, 23). GC can isolate actin, regulate immune and inflammatory responses, bind fatty acids, and control bone development (24, 25, 26). As the main apolipoprotein in chylomicrons and LDL, APOA4 is related to lipid metabolism, insulin resistance, and oxidative stress (27, 28, 29). FTCD plays an important role in inducing the production of ROS, mitochondrial oxidative stress, and tumor metabolism (29, 30). ITIH2 is closely related to inflammation, kidney disease, tumors, and diabetes (31), while FGA is related to chronic inflammation, lipid metabolism, and complications of diabetes (32).

Based on GO and KEGG pathway analysis, these factors are mainly concentrated in pathways related to glucose and lipid metabolism and vasoconstriction and mainly participate in the processes of protein activation cascade, vasoconstriction, and lipid metabolism regulation. They also play physiological roles through pathways related to cholesterol metabolism and vitamin metabolism and absorption, which indicates that fetuin-B is closely related to glucose and lipid metabolism. It has been found that more fetuin-B is secreted during steatosis in liver cells in* in vitro* experiments. A follow-up study among 1140 patients with abdominal obesity revealed that increased pulse wave conduction velocity was significantly related to a high prevalence of NAFLD and fetuin-B, which suggested that fetuin-B might be associated with subclinical atherosclerosis through fat accumulation. In a cross-sectional study, the researchers found that there was a linear relationship between fetuin-B and LDL-C, but no linear relationship with SBP and DBP was found (9), which may be because the research subjects included in the study were patients with coronary heart disease and confounding factors such as secondary hypertension and history of hypertension were not excluded. In contrast, our research subjects were patients whom were newly diagnosed essential hypertension with normal liver function. In another experiment, the researchers found a linear relationship between fetuin-B and DBP (6), which is consistent with the findings of our experiments, but the relationship between fetuin-B and SBP was not clear.

As a traditional cardiovascular risk factor, lipid metabolism disorders are closely related to atherosclerosis, hypertension, and coronary heart disease (33). At present, research has shown that LDL-C is the main substance that leads to vascular endothelial dysfunction. LDL-C can interact with extracellular matrix, causing chronic inflammation and inducing vascular endothelial injury (34). At the same time, lipid metabolism disorders can aggravate oxidative stress, lead to the accumulation of reactive oxygen species (ROS) in the body and oxidize LDL-C to form oxidized LDL (ox-LDL), which participates in the occurrence and development of various cardiovascular diseases. In addition, ox-LDL has cytotoxicity, which can directly aggravate endothelial function damage. Furthermore, macrophages *in vivo* form foam cells after ingesting ox-LDL. Under normal conditions, their ability to ingest and scavenge is in a dynamic balance. However, when the ingesting level exceeds the scavenging level of phagocytes, foam cells burst and release a large number of lysosomal enzymes, which can further induce vascular endothelial injury. Zhu *et al.* found that the content of fetuin-B was related to the severity of coronary artery disease and the LDL-C level and participated in the occurrence and development of coronary artery disease by aggravating endothelial dysfunction.

In addition, our data revealed that fetuin-B was associated with adipokines. ADI regulates blood pressure by promoting the production of NO and inhibiting vasoconstriction, and prevents atherosclerosis through anti-inflammatory effects and reducing ROS production. It also promotes fatty acid oxidation, inhibits lipid synthesis, and reduces blood lipids. ADI is an endogenous bioactive polypeptide secreted by adipocytes that has attracted much attention because of its protective effect against cardiovascular and metabolic diseases (35). *In vitro* experiments showed that the vascular disease of adiponectin gene knockout mice was more serious, and it had the physiological effects of anti-inflammation, anti-steatosis, and anti-fibrosis. Therefore, we speculate that the effect of fetuin-B on the vascular endothelium and metabolism may be partly realized through its connection with ADI (36). Our study demonstrated that there was a difference in serum fetuin-B between patients with essential hypertension and healthy people. Fetuin-B was positively correlated with LDL-C and ET-1 but negatively correlated with ADI. Therefore, we speculated that fetuin-B might be involved in the occurrence and development of essential hypertension by regulating lipid metabolism. However, the reason for the increased serum fetuin-B in patients with essential hypertension is still unclear, and its specific mechanism still needs further study.

Current research shows that the imbalance of ET-1 and NO can reflect vascular endothelial dysfunction. Hypertension is accompanied by chronic vascular inflammation and endothelial dysfunction, which interact with each other. The imbalance between ET-1 and NO produced by vascular endothelial cells can cause endothelial dysfunction, elevated proinflammatory events, as well as increased oxidative stress, thus leading to the development of hypertension. ADI has many effects, such as anti-inflammatory, anti-atherosclerotic, and inhibitory effects against vascular remodeling. Hypoadiponectinemia is a risk factor for many cardiovascular diseases. Our study found that there is a certain relationship of fetuin-B with ET-1 and ADI. However, its specific molecular mechanism still needs to be verified by molecular experiments, animal experiments, and other means. Our experimental system comprehensively studied the relationship between fetuin-B and blood pressure. Furthermore, we found that the incidence of EH increases with higher plasma fetuin-B levels by using multivariate logistic regression. The mechanism of the elevation of circulating fetuin-B in patients with cardiovascular disease is still unclear. Previous research speculated that fetuin-B may be released into the blood by activated platelets. While this study did not allow us to deduce the causal relationship between the elevation of fetuin-B and hypertension, combined with the previous role of fetuin-B in CVDs, we consider that fetuin-B may contribute to high blood pressure by affecting the function and structure of blood vessels. Fetuin-B is a cytokine secreted into the blood by the liver. It widely exists in the human peripheral circulation and has the advantages of easy access, preservation, and detection. Our study found that fetuin-B is associated with the occurrence and development of hypertension. At present, the incidence rate of hypertension is increasing, but awareness of hypertension and its treatment rate are low. As fetuin-B is highly sensitive for detecting hypertension, it may have certain significance for the early intervention of hypertension, or it may become a new biomarker for clinical application.

However, there are still some limitations in our experiments. This was a cross-sectional study of serum fetuin-B and blood lipids, and other related factors, and a causal relationship between these variables cannot be established. Therefore, a large number of prospective studies are needed to determine the correlation between fetuin-B and essential hypertension. Second, the research objects were from a single research center, the sample size was relatively small, and most of them were middle-aged and elderly people; therefore, there was selection bias. In the future, multicenter clinical research with large sample size is needed to verify the conclusion of this experiment. In addition, the sample size of this study was small. If a larger sample size is adopted, various associations may gain statistical significance.

## Supplementary Materials

Supplementary Material

## Declaration of interest

The authors declare that there is no conflict of interest that could be perceived as prejudicing the impartiality of the research reported.

## Funding

This work was supported by research grants from the Natural Science Foundation of Chongqing http://dx.doi.org/10.13039/501100005230 (cstc2020jcyj-msxmX0466, cstc2021jcyj-msxmX0320); Basic Research and Frontier Exploration Grant of Chongqing Yuzhong District Science and Technology committee (20190117); Chongqing medical scientific research project (Joint project of Chongqing Health commission and Science and Technology Bureau) 2021MSXM283, and China Postdoctoral Science Foundation http://dx.doi.org/10.13039/501100002858 funded project (2018M633329) for RL.
