# Supplementary material for: Evaluation of the correlation between fetuin-B levels and essential hypertension: a cross-sectional study
Source: Endocr Connect. 2023 Nov 8;12(12):e230172. doi: 10.1530/EC-23-0172 (PMC10692697; doi:10.1530/EC-23-0172)
Supplement: Supplementary Material [file supplementary_material.pdf]

Table S1. Row Mean Scores and Cochran–Armitage Trend Test of the impact of plasma Fetuin B on EH prevalence

Model adjusted	Fetuin-B	
	$\chi2$	P
Row Mean Scores Test	84.3242	<0.001
Cochran-Armitage Trend Test	-9.1947	<0.001

A **B**

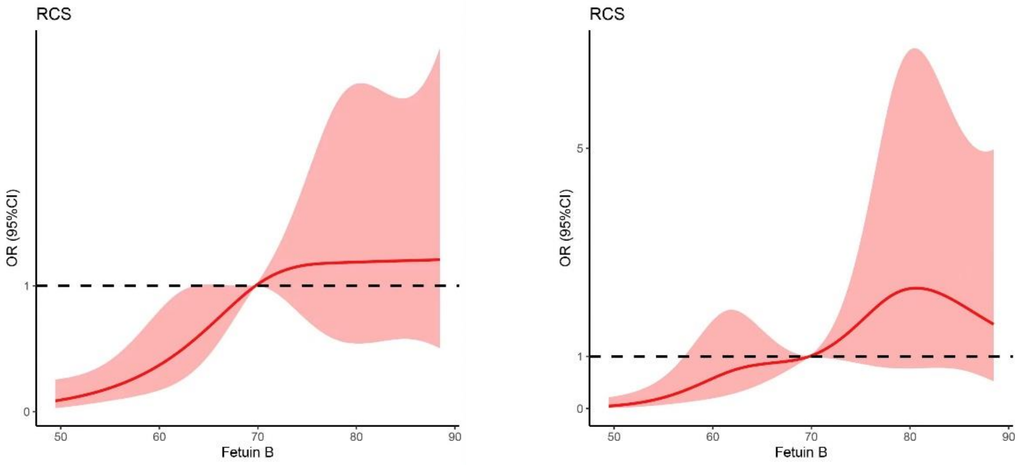

Figure S1. Restricted spline curve of the plasma Fetuin-B level odds ratio of hypertension.

(A)The restricted spline curve of univariable logistic regression model. (B)The restricted spline curve of multivariable logistic regression model. Adjusted for age, gender, BMI, WHR, FAT%, FBG, HbA1c, TG, TC, LDL-C, HDL-C，AST, ALT, GGT, ALP
